# Current advances in biopharmaceutical informatics: guidelines, impact and challenges in the computational developability assessment of antibody therapeutics

**DOI:** 10.1080/19420862.2021.2020082

**Published:** 2022-02-01

**Authors:** Rahul Khetan, Robin Curtis, Charlotte M. Deane, Johannes Thorling Hadsund, Uddipan Kar, Konrad Krawczyk, Daisuke Kuroda, Sarah A. Robinson, Pietro Sormanni, Kouhei Tsumoto, Jim Warwicker, Andrew C.R. Martin

**Affiliations:** a Manchester Institute of Biotechnology, University of Manchester, Manchester, UK; b Department of Statistics, University of Oxford, Oxford, UK; c Department of Mathematics and Computer Science, University of Southern Denmark, Odense, Denmark; d Department of Biological Engineering, Massachusetts Institute of Technology (MIT), Cambridge, MA, USA; e NaturalAntibody, Hamburg, Germany; f Department of Bioengineering, School of Engineering, The University of Tokyo, Tokyo, Japan; g Medical Device Development and Regulation Research Center, School of Engineering, The University of Tokyo, Tokyo, Japan; h Department of Chemistry and Biotechnology, School of Engineering, The University of Tokyo, Tokyo, Japan; i Chemistry of Health, Yusuf Hamied Department of Chemistry, University of Cambridge; j The Institute of Medical Science, The University of Tokyo, Tokyo, Japan; k Institute of Structural and Molecular Biology, Division of Biosciences, University College London, London, UK

**Keywords:** developability guidelines, biopharmaceutical informatics, developability assessment, computational prediction, antibody engineering, therapeutic antibodies

## Abstract

Therapeutic monoclonal antibodies and their derivatives are key components of clinical pipelines in the global biopharmaceutical industry. The availability of large datasets of antibody sequences, structures, and biophysical properties is increasingly enabling the development of predictive models and computational tools for the “developability assessment” of antibody drug candidates. Here, we provide an overview of the antibody informatics tools applicable to the prediction of developability issues such as stability, aggregation, immunogenicity, and chemical degradation. We further evaluate the opportunities and challenges of using biopharmaceutical informatics for drug discovery and optimization. Finally, we discuss the potential of developability guidelines based on *in silico* metrics that can be used for the assessment of antibody stability and manufacturability.

## 1 Introduction

Monoclonal antibodies (mAbs) and antibody-based biotherapeutics represent a unique class of biologics that have greatly reshaped our modern biopharmaceutical industry since the first mAb drug, muromonab (Orthoclone®), was approved by the Food and Drug Administration in June 1986. The global mAb market is currently valued at 152.5 billion USD and is projected to exhibit an annual growth rate of 14.6% in the next decade.^1^ Antibody therapeutics currently in late-stage clinical studies have more than tripled to 88 compared to 2010 and over 550 novel antibody therapeutics are currently in the early-stage commercial clinical pipeline.^1,2^ Antibody therapeutics are anticipated to be the key treatments in a broad range of disease areas, such as cancer, cardiovascular, inflammation, neurological, autoimmune, and infectious diseases.

Biopharmaceutical informatics is the application of computational methods and bioinformatics tools toward addressing challenges in biopharmaceutical drug development. It also includes development of databases containing biophysical data, molecular modeling and simulations, and statistical analysis of biopharmaceutical datasets. The term “Biopharmaceutical Informatics” was first introduced by Kumar *et al*.^3^ as the umbrella term for applications of computational approaches in drug discovery and development. Here, we present different aspects of computational applications to antibody-based biopharmaceutical drug development by highlighting key scientific advances in the developability assessment of antibody-based biologic drug candidates.

One of the first practical applications of software relevant to antibody informatics was the antigenic index,^4^ which was a program to generate surface contour profiles and predict antigenic sites from the linear amino acid sequence of proteins including antibodies. These techniques were the precursors of modern sequence- and structure-based bioinformatics tools used in biopharmaceutical discovery and development. The multitude of computational tools and algorithms now available have ushered in an era of high-throughput biopharmaceutical informatics.

This review is organized into four main sections. The first section outlines the databases and tools available for biopharmaceutical informatics relevant to antibody-based drugs. In the second section, we discuss the role of developability at early-stage development and computational developability assessment of antibody therapeutics. The third section describes the application of biopharmaceutical informatics to identify key developability issues in antibody-based drug discovery and design. The final section summarizes emerging trends in the use of biopharmaceutical informatics for antibody therapeutics. While we discuss antibody informatics tools and approaches for evaluating developability issues, comprehensive review of every developability issue was not possible within this article. We have, however, cited previously published reviews that include more details for each developability issue in the respective sections below.

### 1.1 Creation of databases and data mining for comparison of biophysical attributes

The availability of larger datasets with new high-throughput experimental methods has improved the predictions made by biopharmaceutical informatics tools. The challenge of data scarcity is now being resolved by open-source libraries and public databases of biopharmaceutical data. Data in biopharmaceutical informatics are highly heterogeneous and interrelated. Consequently, it is not possible to capture these broad ranges of properties in a single algorithm. Datasets currently used to assess the biophysical properties of antibodies are curated from internal releases by pharmaceutical companies or data points from scientific papers.^5–7^ Experimental data sourced from scientific papers might not be comparable with one another because of differences in experimental setups, the plethora of developability assays, and different antibody formats tested. Additional data sources that potentially contain much antibody-engineering knowledge are patents, where one needs to scan the documentation for primary sequence information.^8^ Altogether, there is currently much yet-untapped data in the public domain, but these are often hard to curate and not immediately compatible and useful without much earlier pre-processing.

Further advantages from curating antibody databases to learn biophysical properties of antibodies can be obtained by linking information from heterogeneous sources. Current predictive approaches typically use either structural or sequence data rarely linking information from different sources (e.g., structural, and next-generation sequencing (NGS)). Collating information from different sources, however, can augment information available in heterogeneous sources. For instance, structural modeling can provide a conformational dimension to millions of sequences drawn from NGS,^9^ whereas contrasting naturally sourced and therapeutically developed molecules can provide insights on commonalities and divergences between the two sources.^10^ A good example of such an integrated approach is the INDI database,^11^ which contains data for antibody-cognate nanobodies (single-domain antibodies VHH) collected from all major public sources, encompassing patents,^8^ NCBI GenBank, Protein Data Bank (PDB), and NGS/AIRR^12^ supplemented by manual curation from the scientific literature. The sequences and structures of antibodies from these heterogeneous sources are linked with textual information into an antibody-specific database. Integrating the heterogeneous sources in this manner facilitates searching and creation of custom datasets of nanobodies. Extrapolating such data integration approaches to antibodies should allow researchers to focus more on the machine learning/statistical approaches addressing the prediction of biophysical properties of these molecules.

Norman *et al.^13^* have previously provided an overview of available databases and tools for computational antibody analysis. However, our specific focus here is on computational developability assessment tools and databases. Table 1 provides a list of relevant databases and datasets for antibody-based drugs that can be used for training, validation, and assessment of biopharmaceutical informatics tools.

**Table 1.** Relevant databases and datasets for biopharmaceutical informatics

S. No.	Database Name	Application	Link
**Sequence Databases**			
1.	Observed Antibody Space (OAS)	Annotated immune repertoires of over a billion Ab sequences across diverse immune states and organisms.	http://opig.stats.ox.ac.uk/webapps/oas/
2.	International Immunogenetics Information System (IMGT)	IMGT® provides common access to sequence, genome, and structure Immunogenetics data.	http://www.imgt.org/
3.	Patented Antibody Database	The Patented Antibody Database contains sequence information found in patent documents for 267,722 antibody chains from 19,037 patent families.	https://www.naturalantibody.com/pad
4.	iReceptor	Antibody/B-cell and T-cell receptor repertoire data from multiple independent repositories.	https://gateway.ireceptor.org/login
5.	abYsis	Integrated antibody sequence and structure management, analysis, and prediction	http://www.abysis.org/
6.	EMBLIg	Antibody sequences automatically extracted from EMBL-ENA	http://www.abybank.org/emblig/
7.	Antibody Knowledge Graph	A framework for collecting antibody data from all major public sources.	https://www.naturalantibody.com/antibody-knowledge-graph/
8.	Integrated Nanobody Database for Immunoinformatics (INDI)	Database with structure data and sequence information of nanobodies created using an integrated curation approach from several sources.	http://research.naturalantibody.com/nanobodies ^11^
**Structure Databases**			
9.	Protein Data Bank (PDB)	3D structure data for large biological molecules (proteins, DNA, and RNA).	https://www.rcsb.org/
10.	Structural Antibody Database (SAbDab)	An online resource containing all the publicly available antibody structures annotated with several properties.	http://opig.stats.ox.ac.uk/webapps/newsabdab/sabdab/
11.	Thera-SAbDab	Variable domain sequences and structural representations of all antibody therapeutics recognized by the WHO INN lists.	http://opig.stats.ox.ac.uk/webapps/newsabdab/therasabdab/search/ ^14^
12.	SACS	Summary of antibody crystal structures in the PDB	http://www.abybank.org/sacs/
13.	AbDb	Information on redundancy and structures solved with and without antigens for Fv fragments extracted from PDB files.	http://www.abybank.org/abdb/
14.	PyIgClassify	A database of antibody CDR structural classifications	http://dunbrack2.fccc.edu/PyIgClassify/
15.	AAAAA	An automatic modeling and analysis tool for structural alignment of antibody and T cell receptor sequences.	https://plueckthun.bioc.uzh.ch/antibody/index.html
**Immunogenicity**			
16.	Immune Epitope Database (IEDB)	Experimental data on antibody and T cell epitopes.	https://www.iedb.org/
17.	T Cell Epitope Database (TCED™)	Database of CD4+ T cell epitopes derived from T cell epitope mapping studies.	https://abzenaprod.wpengine.com/development-services/immunology/immunogenicity-assessment/itope-and-tced/
18.	MHCBN 4.0	A database of MHC/TAP binding peptides and T-cell epitopes.	http://crdd.osdd.net/raghava/mhcbn/
19.	Bcipep	Database of B-cell epitopes.	https://webs.iiitd.edu.in/raghava/bcipep/info.html
20.	Leadscope Toxicity Database	The Leadscope Toxicity Database contains over 180,000 chemical structures with over 400,000 toxicity study results.	https://www.leadscope.com/product_info.php?products_id=78
**Antibody–antigen binding/Protein–protein interactions**			
21.	PCLICK	Antibody–antigen structures from a dataset of 403 antibody–antigen complexes using CLICK method.	http://mspc.bii.a-star.edu.sg/minhn/cluster_pclick.html
22.	AB-Bind: Antibody binding mutational database	Experimentally determined changes in binding free energies for 1101 mutants across 32 antibody–antigen structures.	https://github.com/sarahsirin/AB-Bind-Database ^15^
23.	SKEMPI 2.0	Database of binding free energy changes upon mutation for structurally resolved protein–protein interactions.	https://life.bsc.es/pid/skempi2/
24.	AntigenDB	Database of antigens from several pathogenic species containing structural, sequence, and binding data	http://crdd.osdd.net/raghava/antigendb/
25.	AntiJen	Database containing quantitative binding data for peptides	http://www.ddg-pharmfac.net/antijen/AntiJen/antijenhomepage.htm
**General Information, Regulatory**			
26.	Tabs – Therapeutic Antibody Database (Commercial-use)	Data on 5,400+ antibodies, 1,350+ antigens, and 1,550+ companies, linked to clinical trials, patents, papers, news, and regulatory agencies.	https://tabs.craic.com/static_pages/4
27.	AbMiner	Database to match commercially available antibodies to their respective genomic identifiers.	https://discover.nci.nih.gov/abminer/

Databases suggested for use in biopharmaceutical informatics relevant for antibody-based drugs. These databases have been selected by authors from several other available databases for general proteins.

### 1.2 Relevance of biopharmaceutical informatics tools

Biopharmaceutical informatics tools are widely used for *in silico* screening of biophysical properties in an antibody library. These antibody informatics approaches have been used to evaluate key biochemical and biophysical properties such as solubility, stability, viscosity, charge profiles, posttranslational modifications (PTMs), pharmacokinetic and pharmacodynamic (PK/PD) profiles, and hydrophobicity to rank the candidates. The prediction of protein tertiary structure is accomplished by either homology modeling approaches, fold recognition, or *ab initio* modeling approaches when similar sequences with known structures are absent. Several studies have implemented homology modeling to calculate the biochemical and biophysical properties of a mAb library.^16–18^ Specific homology modeling algorithms for antibodies have been developed for better accuracy and representation.^19–21^ In general, antibody sequences and structures are well conserved except for the complementarity-determining regions (CDRs). The CDRs, except for CDR-H3, can be classified into a set of limited conformations called canonical structures^22–24^ that can be predicted from sequence key residues, enabling sub-ångström accuracy in structure prediction. However, predicting conformations of CDR-H3 is still challenging because it is the most diverse both in sequence and structure.^25^ Sequence-structure correlations identified for CDR-H3 have been used as geometric constraints in simulations for structure prediction.^26,27^

The antibody modeling tools provide an integrated computer-aided molecular design platform that can be used to access liabilities and optimize the affinity, solubility, and stability of antibody-based drug candidates. Several other biopharmaceutical informatics tools for various developability issues depend on protein sequence features that are based on amino acid physicochemical properties. There have been increasing efforts to compile these tools for integrated antibody sequence and structure management, analysis, and prediction. For instance, a large number of tools for antibody informatics are compiled under the abYsis database, abYmod antibody modeling program, and abYbank database. abYsis^28^ incorporates a wide-ranging species-specific analysis of residue frequencies that can be combined with residue clustering to identify either hydrophobic or unusual patches that are likely to be important for the stability and immunogenicity of antibodies. The Scratch suite of predictors^29^ also provides a set of comprehensive tools to evaluate the physicochemical properties of mAbs, such as the solvent accessibility, secondary structure, tertiary structure, contact maps, protein antigenicity, and domain locations. The Oxford Protein Informatics Group (OPIG) also maintains several webservers and databases relevant to antibody informatics. An up-to-date list of antibody-related resources is maintained at http://naturalantibody.com/tools. Table 2 provides a list of biopharmaceutical informatics tools for the developability assessment of antibody therapeutics.

**Table 2.** Relevant biopharmaceutical informatics tools

Software Name	Application	Link
**Antibody modeling**		
abYmod	Homology modeling, molecular simulations and structural bioinformatics	http://abymod.abysis.org
ABangle	A tool for calculating and analyzing the VH-VL orientation in antibodies.	http://opig.stats.ox.ac.uk/webapps/newsabdab/sabpred/abangle/
ABodyBuilder	Homology modeling, molecular simulations, and structural bioinformatics	http://opig.stats.ox.ac.uk/webapps/abodybuilder
PIGS	Homology modeling, molecular simulations, and structural bioinformatics	https://bio.tools/pigs
MODELLER	Homology modeling, molecular simulations, and structural bioinformatics	https://salilab.org/modeller/
MOE	Homology modeling, molecular simulations, and structural bioinformatics	https://www.chemcomp.com/Products.htm
RosettaAntibody	Homology modeling, molecular simulations, and structural bioinformatics	https://new.rosettacommons.org/docs/latest/application_documentation/antibody/antibody-applications
LYRA	Homology modeling, molecular simulations, and structural bioinformatics	http://www.cbs.dtu.dk/services/LYRA/index.php
Repertoire Builder	Structural modeling of B cell/T cell receptors from their amino acid sequences	https://sysimm.org/rep_builder/
**Solubility and aggregation**		
CamSol	CamSol method constitutes three algorithms to rationally design protein variants with enhanced solubility.	http://www-cohsoftware.ch.cam.ac.uk
Protein-Sol	A web tool for predicting protein solubility from the sequence.	https://protein-sol.manchester.ac.uk/
SODA	Prediction of protein solubility from disorder and aggregation propensity.	http://old.protein.bio.unipd.it/soda/
SOLpro	Protein Solubility predictors	http://scratch.proteomics.ics.uci.edu/explanation.html#SOLpro
SOLart	A structure-based method to predict protein solubility and aggregation using solubility-dependent potentials.	http://babylone.ulb.ac.be/SOLART/
SAP	Aggregation Prediction Spatial aggregation propensity	^30^
Solubis	A webserver to reduce protein aggregation through mutation	http://solubis.switchlab.org/ ^31^
GAP	Aggregation Prediction	https://www.iitm.ac.in/bioinfo/GAP/
AGGRESCAN 3D	Aggregation Prediction	http://bioinf.uab.es/aggrescan/ ^32^
AggScore	Aggregation Prediction	https://www.schrodinger.com/science-articles/aggregation-prediction-protein-surface-analyzer
PASTA 2.0	Aggregation Prediction	http://old.protein.bio.unipd.it/pasta2/
TANGO	Aggregation Prediction	http://tango.crg.es/
**Posttranslational modifications/Stability**		
MusiteDeep	A deep-learning based webserver for protein posttranslational modification site prediction and visualization.	https://github.com/duolinwang/MusiteDeep_web
PTM prediction tools survey	Collection of publicly available PTM web resources, databases, and classification/prediction servers.	http://www.cbs.dtu.dk/databases/PTMpredictions/
MUpro	Prediction of protein stability changes for single-site mutations	http://mupro.proteomics.ics.uci.edu
FindMod	Tool to predict potential protein posttranslational modifications	https://web.expasy.org/findmod/
SIDEpro	Prediction of protein side-chain conformations	http://sidepro.proteomics.ics.uci.edu/
SCWRL4.0	Prediction of protein side-chain conformations	http://dunbrack.fccc.edu/scwrl4/SCWRL4.php
PEARS	Prediction of protein side-chain conformations	http://opig.stats.ox.ac.uk/webapps/pears
**Molecular docking**		
DockThor	Molecular docking, Affinity maturation	https://dockthor.lncc.br/v2/
SwissDock	Molecular docking, Affinity maturation	http://www.swissdock.ch/
HADDOCK	Molecular docking, Affinity maturation	https://wenmr.science.uu.nl/haddock2.4/
MEGADOCK 4.0	Molecular docking, Affinity maturation	https://www.bi.cs.titech.ac.jp/megadock/
RosettaDock	Molecular docking, Affinity maturation	https://new.rosettacommons.org/docs/latest/application_documentation/docking/docking-protocol
FTDock 2.0	Molecular docking, Affinity maturation	http://www.sbg.bio.ic.ac.uk/docking/ftdock.html
AbAdapt	Antibody-specific epitope prediction	https://sysimm.org/abadapt/
**Immunogenicity**		
ANTIGENpro	Protein Antigenicity predictor	http://scratch.proteomics.ics.uci.edu/explanation.html#ANTIGENpro
COBEpro	Continuous B-cell epitope predictor.	http://scratch.proteomics.ics.uci.edu/explanation.html#COBEpro
BEpro (PEPITO)	Discontinuous B-cell epitope predictor.	http://pepito.proteomics.ics.uci.edu
DiscoTope	Prediction of discontinuous B cell epitopes from protein three-dimensional structures	http://www.cbs.dtu.dk/services/DiscoTope/
ElliPro	Antibody epitope prediction	http://tools.iedb.org/ellipro/
SVMTriP	A tool to predict linear antigenic epitopes	http://sysbio.unl.edu/SVMTriP/
AbAdapt	Antibody-specific epitope prediction	https://sysimm.org/abadapt/
EpiPred	Antibody-specific epitope prediction	http://opig.stats.ox.ac.uk/webapps/newsabdab/sabpred/epipred/
RANKPEP	Immunogenicity risk assessment	http://imed.med.ucm.es/Tools/rankpep.html
ProPred	Immunogenicity risk assessment	http://crdd.osdd.net/raghava/propred/
NetMHCIIpan	Immunogenicity risk assessment	http://www.cbs.dtu.dk/services/NetMHCIIpan/
MHCEpitopeEnergy	Rosetta-based biotherapeutic deimmunization platform	https://new.rosettacommons.org/docs/latest/rosetta_basics/scoring/MHCEpitopeEnergy
Hu-mAb	Antibody humanization tool	http://opig.stats.ox.ac.uk/webapps/newsabdab/sabpred/humab
TOPKAT	*in silico* toxicology assessments	https://www.toxit.it/en/services/software/topkat
MetaDrug	*in silico* toxicology assessments	https://support.clarivate.com/LifeSciences/s/article/MetaDrug-Uses-and-benefits?language=en_US
**Biophysical properties**		
Abpred	Prediction of biophysical performance	https://protein-sol.manchester.ac.uk/abpred
QikProp	ADME prediction tool	https://www.schrodinger.com/products/qikprop
Delayed HIC retention time Prediction tool	Model for prediction of delayed HIC retention times directly from sequence.	^33^
**General developability**		
Therapeutic Antibody Profiler (TAP)	Developability guidelines check and Identification of sequence liabilities	http://opig.stats.ox.ac.uk/webapps/newsabdab/sabpred/tap
Developability Index	Developability Index is a function of an antibody’s net charge and the spatial aggregation propensity, calculated on the complementarity-determining region structure.	^34^
abYsis	Integrated antibody sequence and structure management, analysis, and prediction	http://www.abysis.org/
NaturalAntibody AbMapper	A data-driven suite of analytics to improve research decision support in screening and rational design of antibody therapeutics.	https://naturalantibody.com/antibody-analytics/

Biopharmaceutical informatics tools for assessment of developability issues. Most of the tools listed are free for academic use or available on request. Some tools may have an upgraded commercial version for users. These tools have been selected by authors from several other available antibody informatics tools for general proteins.

## 2 Computational developability assessment using biopharmaceutical informatics

Novel criteria based on biochemical and biophysical properties of mAbs are being increasingly used to select a mAb candidate from the early discovery to the development stage. Computational developability assessment approaches are now becoming a routine step in the drug discovery and development process. Developability assessments at the early stage of development can significantly de-risk development pipelines, thus saving valuable time and resources. Incorporating developability assessments in early-stage development provides an opportunity to re-engineer the molecule to mitigate any sequence or structural liabilities, or to select alternative molecules of similar potency, but with more favorable developability profiles. Previous studies have summarized various experimental platforms and computational tools to identify developability issues in therapeutic antibodies and antibody-like molecules.^35,36^

**Figure 1.**
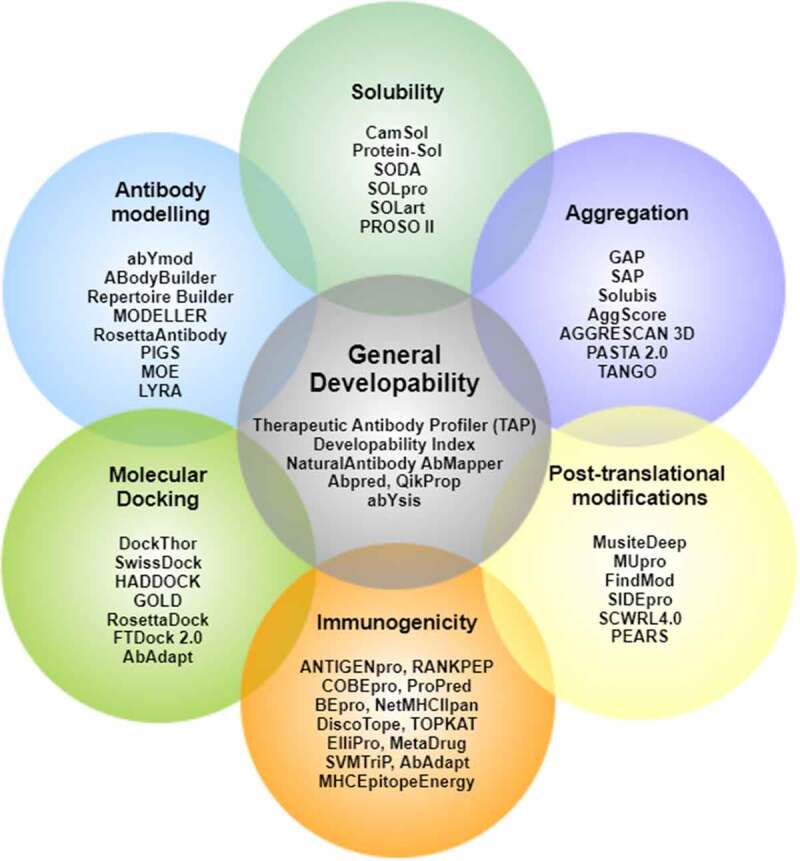
Biopharmaceutical informatics tools for computational developability assessment of antibody therapeutics. These tools have been selected by authors from several other available antibody informatics tools for general proteins. Figure 1. Venn diagram of tools listed under different developability categories.

In the past decade, applications of techniques such as phage display, cell surface display, yeast display, hybridoma, and NGS have revolutionized biomedical research with the successful discovery of several therapeutic antibodies. Although most antibody libraries focus on maximizing library diversity, there are growing concerns regarding the developability of the selected antibodies for successful commercialization.^6^ Therefore, frameworks and procedures are being developed for the design of antibody libraries with improved developability and manufacturability.^37^ *In silico* engineering and design of biologics using rational design principles has emerged as a faster and economic alternative to traditional methods of lead generation such as hybridoma and phage display. Figure 1 provides a visual representation of the recommended biopharmaceutical informatics tools for computational developability assessment of antibody therapeutics and antibody-based drugs.

### 2.1 De-risking biopharmaceutical development using developability assessments

Developability assessment is used to systematically evaluate mAb candidates that have the lowest risks for development to the final product. Previous studies have demonstrated the utility of the developability assessment of mAb lead candidates for screening out mAbs with low solubility and stability, low potency, high aggregation propensity, and high immunogenicity risk.^38^ Any such predictions will inevitably reject some antibodies that could have made excellent drugs, but not using such approaches comes with huge financial risk.

The general biophysical properties of approved mAbs can serve as a reference for the design of new mAb candidates. Several databases of biophysical properties of these approved mAb candidates have been reported such as the Jain dataset^6^ and TheraSabDab.^14^ The Jain dataset provides biophysical characterization across 12 different platforms for 137 clinical-stage and approved antibodies.^6^ This benchmarking with approved mAbs provides an estimate of the acceptable ranges of the biophysical properties that can be considered in the developability assessments for new antibody candidates. Xu *et al*. have outlined some generally preferred quality attributes of a panel of approved and clinical stage mAb products.^39^ The general concept of examining the properties of successful antibody-based drugs has been exploited by Raybould *et al.^5^* resulting in Therapeutic Antibody Profiler (TAP) developability guidelines that are derived from the values of 377 post-Phase 1 clinical-stage antibody therapeutics. It relies on the hypothesis that antibodies that have deviating biophysical properties from clinically tested therapeutic mAbs are likely to have poor developability profiles. TAP can be used to analyze several properties linked to poor developability for any candidate mAb with known heavy and light chain variable domain sequences.

In addition, Abpred^40^ can be used to predict the biophysical performance on 12 commonly used developability assessment assays with just the amino acid sequence input. In Abpred, machine learning methods have been trained on heavy and light chain variable domain sequences from the Jain dataset using the amino acid composition and 15 sequence-derived features to represent physicochemical properties of antibodies. Other developability assessments using machine learning approaches have been used to predict and select the antibodies with optimal pH and thermal stabilities from 77 antibodies in development at Pfizer.^41^ Lonza Biologics has also demonstrated the use of aggregation propensity screening along with other computational approaches during early drug development to select molecules with reduced risk of aggregation and optimal developability properties for screening several anti-interferon γ antibody variants.^7^ Pfizer has implemented *in vitro* assays that correlate with *in vivo* human studies to differentiate mAbs at high risk for rapid clearance from those with favorable PK.^42^ Finally, molecular dynamics simulation has also implemented a high-throughput developability workflow on a panel of 152 human or humanized mAbs.^43^ Here, physicochemical properties of these 152 mAbs were evaluated from multiple biophysical assays – size exclusion chromatography for aggregation, reverse-phase chromatography and sodium dodecyl sulfate capillary electrophoresis for purity, differential scanning fluorimetry for thermostability, hydrophobic interaction chromatography (HIC) for hydrophobicity, affinity-capture self-interaction nanoparticle spectrometry for self-interaction and capillary isoelectric focusing for isoelectric point (pI) and charge variant analysis. These examined biophysical properties and key assay endpoints were also predictive of key downstream process parameters in development and clinical manufacturing.^43^

### 2.2 Design of antibody libraries with improved developability

Screening libraries of antibodies is a commonly used strategy in antibody drug discovery. There are two main approaches to library design: creation of (1) a highly diverse library potentially containing binders to varied targets or (2) a library focused on potential binders to a specific antigen or set of antigens. The ideal library contains genetically varied antibodies with the potential for high affinity and activity, but this can result in the generation of increasingly large libraries to achieve high diversity. With the huge amount of available sequence data and increased understanding of developability prediction, methods are being investigated for the optimal design of antibody libraries with high functionality and desired biophysical properties.

#### 2.2.1 Natural

Methods using B-cell receptor (BCR), i.e., antibody repertoires from antigen-exposed animals or humans (“immune libraries”, to generate antigen-specific libraries) or from non-exposed humans (“naïve libraries”, to generate functionally diverse libraries) try to capture the capabilities of the natural immune response in making functional, highly expressed and low immunogenicity antibodies. However, not all naturally occurring antibodies are suitable drug candidates owing to other developability concerns, such as aggregation.^5^ Libraries can aim to combat this by selecting for genes with known favorable characteristics using native heavy and light chains for improved specificity.^44–46^ Limitations to natural-repertoire approaches also include the inherently biased nature, meaning diverse antibodies may be missed owing to sequence space restrictions. Nevertheless, available sequence space might not be as constrained as previously expected, as multiple clinical-stage therapeutics have high sequence-identity matches in naturally sourced antibody repertoires.^10^ Another way to select antibodies is by considering the “structural space”. For example, a library of antibody structures identified in the repertoires of multiple individuals was found to contain structures highly similar to clinical-stage therapeutic antibodies^47^ and may suggest antibodies with functionality and low likelihood of immunogenicity.

#### 2.2.2 Synthetic

Synthetic libraries introduce diversity, often at defined regions of an antibody, to generate novel and varied sequences. Such methods can produce antibodies with higher affinity than natural repertoires,^48^ but a proportion of the library may be non-folding or immunogenic. To reduce nonfunctionality, methods such as position frequency analysis (PFA) and deep learning have been applied. PFA introduces mutations based on the amino acid frequencies found at each CDR position in natural antibody repertoires, often using identical or only a small number of framework regions.^49,50^ Such methods do not account for correlations between residues at different sites. A different approach has used a database of antibodies with known functionality and interchanged CDR regions, assuming CDR regions are modular and can be interchanged without negative impact. In doing so, they achieved high functionality.^51^

#### 2.2.3 Deep learning

Deep learning models aim to utilize the stability of natural repertoires and capture higher-order dependencies, missed by PFA, to avoid producing nonfunctional proteins. However, current limitations of deep learning approaches include a focus on only CDR regions or heavy chains, with a lack of experimental validation of predicted properties. For instance, 74% antigen binding was achieved in a mouse library designed by a variational autoencoder that generated novel CDR-H regions, but such an approach ignores non-CDR region contributions to the paratope, and the diversity of the sequences in this library is unknown.^52^ Other generative approaches such as Generative Adversarial Networks can be trained on natural human antibody repertoires and biased via transfer learning (further training on antibodies with known properties such as solubility, stability and predicted immunogenicity) to generate sequences predicted to have the desired biophysical properties.^53^ However, more information is needed to understand how such properties influence the overall developability of the antibody. Additionally, experimental validation of the predicted properties is necessary, as has been conducted for an enzymatically active protein library^54^ and a nanobody library created by a generative deep neural network-powered autoregressive model trained on a native llama repertoire.^55^

Previous work has demonstrated the use of mammalian display libraries for the selection of antibody variants with optimal biophysical properties, reduced polyreactivity, and immunogenicity.^56^ Here, they have described the use of a nuclease-directed integration system to generate antibody variants with differing biophysical properties based only on the display level achieved on the mammalian cell surface. Other studies have demonstrated the use of machine learning-guided directed evolution on the combinatorial sequence space.^57^ Recently, a machine learning pipeline has been formulated to predict the developability of a library of 2400 antibodies from sequence alone.^58^ These advances in bioinformatics and *in silico* methods have enabled the efficient development of commercially viable antibodies. Thus, antibody library variants of an antibody candidate are designed to exhibit better developability than the parent molecule.

### 2.3 Mitigating aggregation and post-translational modifications in biopharmaceuticals

#### 2.3.1 Aggregation

Aggregation of antibody-based drugs can lead to precipitation and decreased shelf-life of drugs before administration, while aggregation *in vivo* can increase the immunogenicity of the drug. The aggregation propensity is a critical attribute correlated with product failure.^59^ Indeed, aggregate levels in the final drug product are key quality indicators.^60,61^ Seeliger *et al*. have highlighted four key factors that must be avoided to minimize aggregation, many of which can be predicted computationally: (1) the number of “reactive sites”, such as those susceptible to oxidation, deamidation, or proteolysis, should be minimized; (2) thermodynamic stability should be high to minimize protein unfolding; (3) the structure should not contain hydrophobic or charged surface patches; and (4) the sequence should not contain cross-beta-sheet aggregation hotspots.^62^

Van der Kant *et al*. showed that mutating residues in predicted aggregation hotspots could reduce aggregation and found that those hotspots having the largest impact on thermodynamic instability are frequently found in the CDRs.^63^ The solubility can be improved in mAbs having aggregation-prone regions (APRs) by inserting glycosylation sites near these APRs.^64,65^ Several other studies have used protein-engineering approaches to reduce self-association and aggregation to achieve high solubility and low viscosity.^66–69^ A specific prediction of the tendency to aggregation is the AggScore,^70^ which uses structural modeling to identify patches at risk of driving aggregation. Several methods have been developed to create so-called “developability indices” for antibodies and these tend to focus on aggregation propensity. For example, Lauer *et al*. used data from the storage of 12 IgG antibodies for periods of up to 2 y to examine aggregation. They then combined net charge (at a given pH using a calculated pKa) with a “spatial aggregation propensity” (SAP) score (derived from accessibility and residue hydrophobicity and calculated over a molecular dynamics simulation) to create their developability index and correlated this with the experimental aggregation propensity.^34^ Developability Index^34^ is a well-known tool for estimating the developability of a candidate antibody. However, a potential drawback of the Developability Index is that it is based only on the full-length antibody’s net charge and the SAP of the CDR region, and, therefore, may ignore other indicators of developability.

The Therapeutic Antibody Profiler (TAP) has been demonstrated to be very useful in selectively highlighting antibodies with expression or aggregation issues.^5^ Further, Lonza’s aggregation prediction tool^7^ has been instrumental in the selection of lead antibody candidates from combinatorial libraries with improved developability. abYsis^28^ incorporates a wide-ranging species-specific analysis of residue frequencies that can be combined with residue clustering to identify either hydrophobic or unusual patches that are likely to be important for the stability and immunogenicity of biopharmaceuticals. Therefore, using these computational aggregation prediction tools can identify aggregation issues early in biopharmaceutical development and avoid expensive late-stage product failures.

### 2.3.2 Post-translational modifications

PTMs can lead to several issues encountered with the development of antibodies. By their nature, PTMs lead to heterogeneity, something that generally concerns regulators since variants must be considered in risk assessments and during characterization to assess the impact on product quality, safety, and efficacy. This includes potential effects on antigen binding, immunogenicity, and Fc-mediated effector functions.

In antibodies, the N-terminal glutamate or glutamine is frequently cyclized by nucleophilic attack of the lone pair of electrons from the backbone terminal NH2 onto the sidechain carboxy or amide, forming a five-membered lactam ring known variously as pyroglutamic acid (pyroGlu), pyrrolidone carboxylic acid (PCA), 5-oxoproline, or pidolic acid, and this has been shown to occur *in vitro*.^71–73^ The N-terminus is comparatively close to the antigen binding site, so the difference in charge could have an effect on antigen binding, particularly for large antigens that may approach close to this part of the antibody. In addition to N-terminal heterogeneity, “clipping” frequently occurs at the C-terminus of the heavy chain. The last three residues of the heavy chain are Pro-Gly-Lys; the proline is the last residue of the CH3 domain, and the glycine and lysine form the CHS region. The C-terminal lysine is mostly clipped posttranslationally by endogenous carboxypeptidases during cell culture, or by endogenous serum carboxypeptidase B once the antibody is administered to a patient.^74^ However, this PTM is unlikely to have any serious effect on the *in vivo* performance of antibody-based drugs since the C-terminus is remote from any functional sites. That said, C-terminal clipping has been shown to be required for optimal complement activation and the presence of the lysine can affect the blood circulation time.^75^ The third major PTM in antibodies is the N-linked glycosylation present in the CH2 domain. While these are the three best-known PTMs present in the vast majority of antibodies, many other sequence-specific PTMs are also observed, all of which lead to heterogeneity potentially affecting charge, pI, aggregation, and binding. Heterogeneity as a result of PTMs and their effects are reviewed by Liu *et al*.,^76^ while a comprehensive analysis of charge heterogeneity in adalimumab (Humira®) was performed by Füssl *et al*.^77^

Asparagine and aspartate residues form hot spots susceptible to deamidation and isomerization.^39,78^ In addition to the effect of antibody deamidation, there have been reports of deamidation in protein antigens in severe diseases such as anthrax.^79^ Oxidation of methionine and tryptophan residues is another sequence liability that can lead to low potency, decreased thermal stability, and high aggregation propensity.^80,81^ Disulfide scrambling due to cysteine residues is another phenomenon causing configurational changes in the hinge region of antibodies, thus impeding antigen binding and mAb functionalities.^82,83^ The variable domains of mAbs may also contain N-glycosylation sites, which may cause variable domain glycosylation that results in the formation of Fab-associated oligosaccharides with α1,3-galactose that are known to cause immunogenicity.^84–86^ These PTMs often lead to low potency, immunogenicity, and instability of circulating mAbs.^87^ Consequently, suitable developability assessment protocols must be designed to capture these sequence liabilities.

abYsis^28^ (http://www.abysis.org) provides screens for a number of these PTMs for optimization of therapeutic antibodies. It also annotates residues as being exposed, buried, or intermediate based on averaged information from several hundred known structures and can be used in concert with abYmod (http://abymod.abysis.org) to build an antibody model from which more detailed exposure information can be obtained. As described, PTMs could seriously hamper the safety or efficacy of therapeutic antibodies and this safety concern calls for an immediate need for appropriate tools to relate a biophysical property to a single, or a set of, molecular sequence-structural motifs in biologic drugs. In summary, biopharmaceutical informatics tools are used to locate the amino acids critical for certain biophysical properties that are in undesirable ranges.

### 2.4 *Biopharmaceutical informatics for drug safety and* in vivo *performance*

#### 2.4.1 Drug safety

A strategic framework for using computational tools for predicting chemical degradation sites in biologic drugs has been presented in a previous study by Sandeep *et al.^3^* Several computational tools for predicting the toxicity of antibody-based drugs are now available.^88^ A critically important step in drug development for establishing clinical safety is the identification of adverse drug reactions (ADRs). Computer-aided prediction of ADRs provides an alternative to recognize ADRs before clinical trials. Kuang *et al*. have reviewed and compared the computational models available for predicting ADRs.^89^ Here, among the topological features of drug-ADR association networks, the Jaccard coefficient (a measure of the relationship between the neighborhood set of homology nodes) was the most important feature for the prediction of drug-ADR associations. Consequently, the Jaccard coefficient of drug-ADR association networks is an important topological feature that should be used in models designed for prediction of antibody drug safety.

Previous computational approaches have estimated *in vivo* performance descriptors such as the PK, PD, and immunogenicity of biologics.^42,90–93^ Avery *et al*. have demonstrated a combinatorial triage approach on *in vitro* assay parameters and categories for screening therapeutic mAb candidates with desirable PK properties and minimal non-target-related PK risk.^42^ Here, threshold values of *in vitro* assays reflecting nonspecific interactions and self-association were established to define criteria for avoiding the selection of mAbs with rapid *in vivo* clearance. Grinshpun *et al*. have also analyzed biophysical and sequence-based *in silico* properties that are predictive of PK properties such as clearance for a panel of 64 clinical-stage mAbs.^94^ They have concluded that experimental poly-specificity assay results and *in silico* estimated pIs were the best predictors to estimate clearance in therapeutic antibodies.

### 2.4.2 Antigen–antibody interactions

General protein–protein interaction prediction tools for proteins frequently do not work well for antigen–antibody interactions because antibody–antigen binding is a rather distinct mechanism. Unlike normal protein interfaces, the epitope on an antigen has evolved to be an exposed region rather than to be involved in a protein–protein interface. Consequently, other computational techniques such as epitope mapping are used to identify the regions of an antigen likely to form the epitope before docking. B-cell Epitope (BCE) mapping tools can broadly be divided into linear epitope predictors, which attempt to identify epitopes consisting of continuous amino acid primary sequences, and conformational epitope predictors, predicting discontinuous epitopes in three-dimensional (3D) space.^13^ However, like other protein–protein interfaces, antibody–antigen interactions involve a combination of nonpolar van der Waals interactions, hydrogen bonding, charge interactions, and the hydrophobic effect. Consequently, along with these epitope prediction tools, several docking algorithms such as Megadock, Haddock, RosettaDock, and Piper are being actively used to understand the binding between an antibody and the target. However, their performance is often poor compared with general protein–protein docking.

#### 2.4.3 Immunogenicity

The presence of T-cell and B-cell epitopes influences the immunogenicity of antibody therapeutics, and, therefore, bioinformatics approaches to avoid immunogenicity fall into two major categories: T-cell epitope prediction and B-cell epitope prediction. Computational tools for immunogenicity risk assessment provide an alternative to *in vitro* or *in vivo* immunogenicity assays. The use of *in silico* tools to identify lead candidates with a reduced risk of immunogenicity is an important step in biologic drug development.

T-cell epitope prediction, which is relatively well established, requires predicting linear peptides within a protein sequence that will bind to the Major Histocompatibility Complex (MHC). MHC molecules present peptides to T cells, which trigger T-cell immune responses. MHC molecules can be classified into class I and class II. MHC class I molecules present peptides derived from intracellular proteins, whereas MHC class II presents peptides from extracellular proteins. Since antibodies are extracellular, the focus is on the prediction of peptide binding to MHC class II molecules. These tools usually examine the primary sequences of candidate antibodies to identify binding motifs of MHC class II allotypes or for similarity to epitopes known to elicit an immune response. Several MHC class II binding predictors are available and the overall prediction performance is generally good.^95,96^

For example, some tools such as RANKPEP,^97^ Propred,^98^ Tepitope,^99^ and NetMHCII^100^ make predictions based on algorithms trained on MHC class II binding assay data. Other tools such as NetMHCIIpan and IEDB (Consensus)^101^ are based on sequence alignments with MHC class II binding peptide databases. Overall, studies have established that NetMHCIIpan, Propred, IEDB (Consensus), and MULTIPRED^102^ were the best predictors of MHC class II binding and these are the most commonly used tools in the industry for the prediction of MHC class II binding. Other previous studies compared nine different MHC class II binding prediction tools and six different methods showing that NetMHCIIpan was the best method to predict peptide binding to MHC class II epitopes with an updated version, having improved predictions, now available.^103,104^ While less important for antibody-based drugs, computational tools for determining binding to MHC class I molecules require locating motifs that bind to the binding groove. Prediction methods for interrogating peptide binding to MHC class I alleles include NetMHC-3.0,^105^ NetMHCpan-1.0, the Kernel-based Inter-allele peptide binding prediction system,^106^ and Adaptive Double Threading.^107^ Based on this predicted T-cell epitope information, Yachnin *et al*. recently developed a Rosetta-based platform to deimmunize therapeutic proteins.^108^ They incorporated a new score term utilizing predicted or experimentally identified T-cell epitope information into the scoring function so that computational protein design calculations can be guided based on the epitope information as well as the energetic stability.

In contrast to the prediction of T-cell epitopes, a much harder task is B-cell epitope (BCE) prediction – predicting sites where the patient antibodies will bind to the drug. Such approaches have not been very successful, mostly owing to the discontinuity of antigen binding sites. As mentioned above, the problem is made harder by the fact that B-cell epitopes are, by their nature, regions of a protein surface that have not evolved to be involved in protein–protein interactions. Consequently, they do not have clearly recognizable features that are bound by antibodies.^109^ Nonetheless, some regions will be more likely to interact with an antibody than others, but making mutations to remove a dominant B-cell epitope can simply result in the immune response switching to a less dominant epitope.

Several predictors have been produced that work at either the sequence level or the level of 3D structure. The earliest BCE prediction methods attempted to predict linear epitopes (i.e., a continuous stretch of amino acid sequence) using sequence features such as hydrophilicity,^110^ amino acid composition,^111^ and predicted accessibility and mobility.^112^ An early evaluation showed that no single sequence feature performed well, leading to attempts to combine features.^113^ However, machine learning efforts^114^ and additional features such as sequence conservation^115^ have provided limited improvements to BCE prediction. In general, conformational epitope predictors such as CBTOPE, BETOPE, CEP, and DISCOTOPE are more accurate than linear epitope predictors such as LBTope, SYMTriP, and ABCored.^116–118^

The performance of computational epitope prediction tools and tools for predicting immunogenicity has been reviewed previously to establish guidelines for the deimmunization of protein therapeutics.^119^ It is worth nothing that epitope databases are not exhaustive because of the heterogeneity of proteins involved in the immune response across the human population.^90^ This variability of immune response for the same antigen limits the utility of *in silico* immunogenicity assessment methods as stand-alone tools. Therefore, this key limitation of immune response diversity needs to be captured by the forthcoming immunogenicity prediction tools.

### 2.5 Guidelines for the design of developability assessment protocols

Assessment of developability by biopharmaceutical informatics protocols at an early stage in a development pipeline reduces the costs of development failures. Companies using transgenic mice to produce antibodies can generate as many as a million sequences a week (after cleaning the high-throughput sequence data) and it is impractical to take all these through to experimental validation. Even computational evaluation requires significant computing resources and optimization. If each sequence takes 1 s to analyze, a million sequences will require ~11.5 days of computer time. Consequently, it makes sense to use a triaging pipeline that performs evaluations that can be done quickly first and leave more computer-intensive evaluations to be performed only on those sequences that have survived the initial rapid triages.

**Figure 2.**
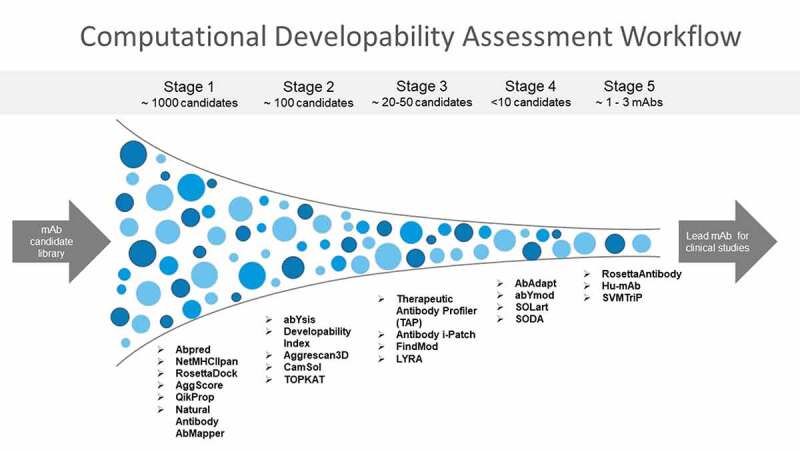
Computational developability assessment workflow for screening mAbs with optimal biophysical properties. An orthogonal combination of conceptually different algorithms is used to reduce method-specific biases. High-throughput antibody informatics tools are implemented first to an antibody library. mAbs scoring above assay thresholds or having results outside the acceptable range are deprioritized. Next, more computationally intensive antibody informatics tools are applied to evaluate additional developability issues. The final step in the CDA workflow is to use a combinatorial triage approach to combine scores and rankings from multiple tools together and classify the mAbs based on the aggregate result of all tools. Figure 2. Funnel flowchart with mAbs shown in blue dots and tools listed for each stage of developability assessment.

A screening paradigm used in the industry for selecting mAbs with desirable PK properties during mAb discovery and lead selection has been demonstrated in a previous study.^42^ This staged approach for developability assessment involves using the high-throughput assays first when hundreds of mAbs are available for screening. Here, mAbs scoring above assay thresholds or having results outside the acceptable range are deprioritized because they have unfavorable physicochemical properties. Next, additional physicochemical properties such as thermal stability are evaluated for only the mAbs that have passed the previous stage. These additional screens include assays measuring properties such as biological activity, expression, stability that are often low-throughput and need higher quantities of mAbs. Finally, a combinatorial triage approach is used that ranks and classifies the mAbs based on the aggregate result of all the assays. It is very important to combine results of multiple assays together since individual developability assays can have some false-positive results. This ensures that mAbs with desirable physicochemical properties advance to scale-up and costly preclinical and clinical development. A Computational Developability Assessment (CDA) workflow should follow a similar strategy where a panel of high-throughput computationally undemanding tools is applied first to a mAb library followed by specific computationally intensive antibody informatics tools as per the required objective, such as those for immunogenicity assessment. The final step in the CDA workflow as shown in Figure 2 is to use a combinatorial triage approach to combine scores and rankings from multiple tools together and classify the mAbs based on the aggregate result of all the informatics tools.

Together with previously discussed approaches to assessing developability, Raybould *et al*. have described five computational developability guidelines for therapeutic antibody profiling: (1) total CDR length, (2) patches of surface hydrophobicity (PSH) metric across the CDR vicinity, (3) patches of positive charge (PPC) metric across the CDR vicinity, (4) patches of negative charge (PNC) metric across the CDR vicinity, and (5) structural Fv charge symmetry parameter.^5^ Overall, local charge and global charge asymmetry between the CDR and the framework have been correlated with higher aggregation and poor developability. Here, the approach was to look at the characteristics of clinically successful antibodies and rank candidate antibodies by ensuring they stay within these bounds. This is conceptually similar to Lipinski’s rules used in small-molecule drug design.^120^ An efficient high-throughput developability workflow was also demonstrated by Bailly *et al*. on a panel of 152 mAbs for rank ordering of molecules during early-stage discovery screening.^43^ Here, they have demonstrated that key physicochemical properties from multiple biophysical assays correlated well with major downstream process parameters.

As above, most types of analysis performed for developability assessment include identification of PTM sites, analysis of likely aggregation propensity (largely through examining surface hydrophobicity), pI, prediction of stability, and identification of T-cell epitopes/B-cell epitopes together with humanness scoring or unusual surface patches. Other considerations that can be included early in the pipeline (as they are fast to evaluate) include checking for the presence of the standard two cysteines present in antibody variable domains, the Trp-Gly motif present immediately after CDR-H3, and the length of CDR-H3 since unusually long CDR-H3 loops have been correlated with poor developability.^121^ However, each tool relies on different interpretations and weighting of the essential features that determine developability. Therefore, an orthogonal combination of conceptually different algorithms should be used in computational developability assessment protocols to reduce method-specific biases.

## 3 Applications of biopharmaceutical informatics

### 3.1 Biopharmaceutical informatics for solubility predictions

Solubility is one of the key biophysical properties that underpins developability potential, as high solubility typically translates into high expression yields, low aggregation, and provides the opportunity of formulating products at high concentrations while retaining a good shelf life.

Identifying antibodies with low solubility and high aggregation propensity from combinatorial libraries remains a hurdle for antibody development. Several *in silico* predictors have been reported that are now able to predict solubility or aggregation propensity accurately in many cases, a feature that makes them highly competitive with experiments.^37,122–124^ These solubility predictors include CamSol, Protein-Sol, SOLpro, SODA, Aggrescan, SAP, and Solubis. They have been shown to be effective at predicting solubility and aggregation propensity of diverse antibody libraries.^122,124^

As an example, the CamSol method of predicting solubility relies on a combination of physicochemical properties of amino acids. These include charge, hydrophobicity, and propensity to form secondary structure elements, which are first considered at the individual residue level, then averaged locally across sequence regions, and finally considered globally to yield a solubility score.^124,125^ In particular, while a structural model is necessary to identify aggregation hotspots, the solubility prediction itself is performed using only the amino acid sequence. This aspect makes computational calculation significantly faster and makes the method readily applicable to the screening of antibody libraries without the need for structural modeling, and thus it is fully independent of model accuracy. For example, CamSol was used to rank the solubility of hits from a phage-display library from MedImmune.^125^ The mAbs that were analyzed differed by up to 32 mutations in the Fv region, and the correlation between prediction and experiments of PEG-precipitation was R ~ 0.97 after one outlier was removed (p < 10^–4^), which is fully consistent with the R ~ 0.98 reported for a nanobody in the original report.^124^ Similarly, a statistically significant correlation (R ~ 0.71 to 0.93) between CamSol predictions and solubility measurements was also reported for mutational variants of a troublesome mAb.^126^ In a study on a library of 17 mAbs from Novo Nordisk, CamSol predictions were compared with a battery of commonly used developability assays and one measurement of relative solubility, and the correlations between CamSol and these experimental readouts were on a par with those seen between the assays.^127^ Notably, all these measurements were carried out with different experimental techniques, on widely different molecules, and in different laboratories. Taken together, these strong correlations suggest that CamSol predictions can greatly facilitate the screening of solubility and hence of developability potential. In particular, at the initial stages of antibody discovery campaigns, when numbers of candidates can be very high while yield and purity are often low, such predictions may entirely replace experiments.

Kingsbury *et al*. have previously predicted the solution behavior of a diverse dataset of 59 mAbs, including 43 approved antibodies, using a comprehensive array of 23 molecular descriptors categorized as colloidal, electrostatic, conformational, hydrodynamic, and hydrophobic.^128^ They have shown that the diffusion interaction parameter (k_D_), a measure of colloidal self-interaction is the key parameter that is most predictive of solution viscosity and opalescence for mAbs. So, they have postulated that computational developability assessment protocols should use a threshold value of the diffusion interaction parameter, k_D_ (10 mM histidine-HCl buffer at pH 6.0) to screen antibodies with optimal antibody solution behavior.

### 3.2 Biopharmaceutical informatics for predicting protein stability and interactions

There can be opportunities to address the underlying balance of biophysical forces that drive interactions when developing models to predict the properties of biopharmaceutical candidates. Two such examples are discussed here, one relating to the measurement of hydrophobic interactions and the other to the protein structural basis of hydrophobic interaction between proteins. Several machine learning methods to predict the HIC retention time from antibody sequence input have been reported previously in the literature.^33,40,129^ Assessment of aggregation propensity using HIC was the best-predicted biophysical property across 12 models produced using Abpred (www.protein-sol.manchester.ac.uk/abpred), one for each of the 12 biophysical properties measured across a set of antibodies.^40^ Even so, there was a marked reduction in performance of the model for antibodies with higher retention times in HIC, leading to a model in which the salt gradient that is used to modulate hydrophobic interaction strength also affects interactions between charged proteins. A revised scheme was derived in which charge interactions play a role alongside hydrophobic effects in the HIC method. In this scheme, proteins with higher net charge repel more within the column when salt concentration (ionic strength) is lower, and are eluted faster, than proteins with lower net charge but the same hydrophobicity.

In this second example, another set of HIC data for 24 antibodies was used.^130^ Here, aromatic sidechain content of CDRs correlated well with the experimental data, but the equivalent correlation was much lower for the solvent-accessible surface area calculated for nonpolar atoms in the CDRs^131^ and it was concluded that hydrophobic interaction strength may be dependent on nonpolar surface shape as well as surface area, consistent with thermodynamic measurements made for mutations in an antibody–antigen interface.^132^ These examples demonstrate that models rooted in biophysical descriptions of protein stability and interactions, and benchmarked against experimental data, can both provide predictive insight for biopharmaceuticals and further the understanding of the underlying biophysical mechanisms.

### 3.3 Biopharmaceutical informatics for preclinical immunogenicity risk assessment

A key concern with any biologic drug is immunogenicity, the effects of which range from simply having an immune response, meaning that the drug is rapidly cleared from the body when administered, through to the possibility of anaphylactic shock. As described above, methods can be applied to predict T-cell epitopes and (to some extent) B-cell epitopes, but a more practical approach has been to ensure that antibody-based drugs are as human as possible and this has become one of the main aims in producing antibody-based drugs. As described earlier, the first monoclonal antibody-based drug to be approved was a mouse antibody (muromonab). However, since then, efforts have gone into making antibody-based drugs less immunogenic, first by producing chimerics (where the variable domains are from the donor species while constant domains are human) and then by “humanization” (where the CDR loops that form the antibody combining site are from the donor species and the rest of the variable domains is predominantly human, as well as human constant domains).^133^ A halfway step between chimerics and humanization to reduce immunogenicity has been “resurfacing” of chimeric antibodies in which surface residues of the variable domain, away from the CDRs, are mutated to human residues.^134^ This is done to remove primarily B-cell epitopes on the antibody surface. Many antibody-based drugs are now “fully human”, being produced by phage display, using transgenic mice, or by identifying antibodies from recovering patients. However, antibodies produced by such methods can still be immunogenic. For example, adalimumab (Humira®, the world’s top-grossing drug), while “fully human” (produced by guided phage display), elicits an immune response in >25% of patients with only 4% of these patients having sustained remission, compared with 34% of patients who did not have antibodies against adalimumab.^135^

Thus, even with fully human antibodies, computational BCE and TCE predictors can be used to predict B-cell epitopes and T-cell epitopes, which can also be experimentally identified through proteomic assays.^136,137^ It is then desirable to remove these potentially immunogenic regions in advance of clinical trials. As well as the application of BCE and TCE predictors, various “humanness” scores have been proposed based on sequence information of human antibodies, enabling the *in silico* assessment of human-likeness given sequences of antibodies.^138–140^ Recently, Schmitz *et al*. developed a computational method that maps the sequence of a given antibody onto human B-cell repertoires comprising 326 million sequences of human antibodies.^141^ Chin *et al*. built a machine learning-based predictive model that distinguishes human antibody sequences from non-human ones, which was trained on large-scale repertoire dataset.^142^ These human-likeness scoring approaches will be useful when assessing how much given antibodies are close to human repertoires; the more human-like antibodies are, the less immunogenic they are expected to be. As described above, another approach is to identify patches of unusual residues on the protein surface that may lead to an immune response.

## 4 Future perspectives in biopharmaceutical informatics

### 4.1 Decoding human antibody gene repertoires and their role in target validation and drug discovery

New high-throughput sequencing methods have generated a vast amount of antibody sequence data, with over one billion antibody sequences publicly accessible in repositories.^12,28,143–148^ A sequenced human B-cell receptor (BCR, i.e., antibody) repertoire provides a snapshot of the BCRs present, typically those circulating in the blood, at a given time. BCR sequence and structure datasets can be used to investigate immune system mechanisms for improved library design, understand disease pathogenesis and identify antibodies for potential therapeutic development.^149^

#### 4.1.1 Immune system mechanisms

The diversity of BCR repertoires can be used to develop an understanding of the mechanisms underlying the immune system. Typical BCR repertoire profiling includes sequence-based analysis, such as clonotyping. Clonotyping involves clustering sequences into clones, usually based on identical V and J genes and high CDR-H3 identity.^150^ Such analysis can reveal dominant antibody sequences, potentially indicative of a response to an antigen, e.g., after vaccination. The availability of large datasets has been useful in characterizing the response to antigens and estimating true antibody genetic diversity,^151^ though these are still far from fully understood. Sequence-based analysis has revealed that the immune systems of unrelated individuals have similarities; an estimated 0.02% of clones are “public” – shared across multiple individuals.^152^ However, differences identified between identical twins indicate the complexity of the immune response and the importance of epigenetics and environmental factors.^153^ Understanding such mechanisms is useful for antibody drug development, for example, to design antibody libraries for drug discovery.

#### 4.1.2 Understanding disease pathogenesis

Immune responses to disease, and also therapies, can be profiled using BCR repertoires to investigate B cell subtype involvement and levels of antibody response. Using such analysis, we can distinguish between healthy and disease repertoires and learn about disease mechanisms, particularly those associated with B cells, such as autoimmune diseases, chronic lymphoid leukemia, and other cancers.^154,155^ In the future, such information will hopefully be used to improve patient outcomes by identifying the most at-risk patients, tracking disease progression and monitoring response to therapies. A better understanding of the immune system involvement in disease may also indicate targets for potential therapeutic intervention, and even suggest antibody drug candidates present in the BCR repertoires of patients with the disease.

#### 4.1.3 Therapeutic antibody candidate identification – using sequence information

BCR sequence repertoires can be used to suggest suitable candidates for drug development. A previous study has contextualized the sequence and structural properties of clinical-stage antibodies with human immunoglobulin datasets (Ig-seq) to evaluate the extent of humanness/originality of antibodies in clinical investigation.^5^ While not all naturally occurring antibodies make good drug candidates, 29 clinical-stage therapeutic antibodies were found to share 100% CDR-H3 identity with a BCR sequence from a healthy human repertoire.^10,152^ By looking for antibody sequences frequently found after exposure to an antigen, we can identify those that might bind specifically to that particular antigen. When assessing individuals with the same disease or who have been exposed to the same antigen (either through infection or vaccination), these sequence-convergent responses can be a useful starting point for a potential therapeutic. Evidence to support this approach for drug discovery comes from vaccine studies^156^ and more recently SARS-CoV-2-infected individuals, where convergent antibodies had sequence similarity with identified SARS-CoV-2-binding antibodies.^157^ In addition to being potential binders, public clones may also have low immunogenicity, making them attractive as drug candidates.^47^

If existing binders are already known, likely drug candidates can be identified from a BCR repertoire by comparing with known antibodies binding to the desired antigen. Identification can be based on sequence identity, such as clonotyping,^158^ or prediction of similar binding properties.^159^ As such, sequence data from BCR repertoires can be useful starting points for suggested therapeutic antibody candidates, with or without knowledge of existing binding antibodies.

#### 4.1.4 Therapeutic antibody candidate identification – incorporating structural information

While most examination of immune repertoires focuses on sequence analysis, utilizing available structural information may also be important when identifying potential therapeutic antibody candidates. Conventional antibody modeling tools are inefficient for building 3D models of entire repertoires of BCRs, with the fastest taking seconds per antibody model via homology modeling methods^20,160^ or ~285 CPU hours^161^ with *ab initio* methods. Therefore, structural modeling methods have been developed specifically for large-scale BCR or TCR repertoire data analysis. Incorporating structural information from models can allow prediction of antibody properties in a repertoire and we may be able to predict antibody domain binding by performing structural clustering of antibody models with known-function antibody datasets, such as CoV-AbDab.^162,163^

A high-throughput alternative to modeling utilizes structural annotation to rapidly predict antibody CDR loop shapes, based on sequence identity matching to a template.^164^ Repertoires can be evaluated based on predicted CDR structures, for example, to identify over-represented CDR-H3 templates or clusters of templates that may represent a response to an antigen, and therefore be a useful starting point in therapeutic antibody design. Using structural prediction tools with BCR repertoire sequence data can reveal antibody drug candidates not seen using sequence-only analysis.

Current limitations for utilizing BCR repertoire data in drug development include the major challenges of predicting antibody–antigen binding and affinity. In addition, existing BCR sequencing datasets often contain only heavy chain information, and methods for obtaining BCR repertoires and binding affinities are varied and lack standardized protocols and analysis pipelines. With the development of high throughput methods for single-cell sequencing and antigen specificity mapping,^165^ increased amounts of high quality, antigen-labeled antibody data might enable new accurate and reliable computational methods for drug discovery.

### 4.2 Biopharmaceutical informatics for design and optimization of next-generation biotherapeutics

The spectrum of biological activities accessible to antibody therapeutics is being expanded by exploring novel mechanisms of action. For example, bispecific antibodies can be created by engineering different specificities into each arm of the antibody, and multi-specific antibodies can be created by adding further VH/VL domains on the heavy and light chains or as a single-chain Fv (scFv) appended on the N- or C-terminus. In addition, novel binding functions can be created using scFvs or nanobodies (heavy-chain only), often combined in tandem for higher avidity or multi-specificity. Other technologies include antibody–drug conjugates (ADCs) created by conjugating cytotoxic drugs (payloads) for site-specific delivery. These novel antibody constructs are often collectively referred to as “next-generation antibodies”^166^ and are emerging as potential therapeutics with unique properties.

The sequences of these antibody formats may differ substantially from those of immune-system-derived immunoglobulins, as extensive engineering is typically required to bring about the desired functionality. It is often the case that engineering additional functionality comes at the expense of other important properties that underpin developability, including conformational and colloidal stability, solubility, immunogenicity, and PK. Therefore, the successful development of next-generation biotherapeutics presents additional challenges, which are usually system-specific. For example, ADCs are complex molecules that require careful attention to various components, including the mAb, the engineered drug conjugation sites, the selected linker, the payload, and the drug load distribution.^167–169^ Similarly, multi-specific antibodies require the selection of multiple binding domains that must be successfully combined to ultimately yield a homogeneous product with the desired functionality and suitable developability profile.^166^

In general, the computational prediction of the developability potential of these novel antibody-based formats presents two overarching challenges. The first is that there is no guarantee that combining together components with suitable properties will translate into a final therapeutic that has desirable characteristics. For example, a bispecific antibody obtained by combining two Fvs with good developability profiles may present unexpected liabilities, such as increased oligomerization brought about by cross interactions between its components. Therefore, while the tools described here may be used to pre-select or engineer binding domains and mAbs with optimal characteristics, when these are combined in a multi-specific format the resulting construct may not necessarily be well behaved. The second challenge lies in the combinatorial nature of combining multiple constructs, which amplifies prediction errors and hence the risk of failure, even assuming that different components behave independently. As an example, consider a computational predictor of a “good” characteristic (such as having good solubility) with precision, or “positive predictive value” (PPV) of 0.9 that implies a false discovery rate (FDR = 1-PPV) of 0.1 (i.e., of the positive predictions, 90% of them are correct, or in other words, one in ten antibodies that are predicted as good are actually poor). If we apply this method to select two distinct Fvs for a bispecific antibody, then the probability of introducing at least one liability in this construct is given by 1-(PPV),^2^ i.e., 0.19 or 19%. Similarly, for a tri-specific construct, such as nanobodies in tandem, the probability of introducing a “poor” binding domain becomes 27.1%. Therefore, even when neglecting the first challenge and considering the different components as fully independent of each other, the accurate prediction of the developability profile of next-generation biotherapeutics will require exceedingly precise methods.

Some of the databases that can be used for the analysis of nanobody-derived therapeutics are the Single Domain Antibody Database^170^ (sdAb-DB), Integrated Nanobody Database for Immunoinformatics^11^ (INDI Nanobodies DB), Non-redundant Nanobody database^171^ and database of Institute Collection and Analysis of Nanobodies^172^ (iCAN). These databases host large collections of natural and synthetic camelid single-domain antibody sequences from literature sources and other online repositories. Each of these databases further provides unified annotation and integrative analysis tools for describing various single-domain antibodies. Overall, computational predictions of developability potential can already be used to aid the development of next-generation biotherapeutics. However, further developments are required before these methods will become highly competitive with experimental readouts in terms of accuracy and reliability. To accelerate innovation in this area, it will be essential that experimental data of developability are published together with the antibody sequences used in the experiments, including any engineered modifications. We anticipate that, just as the Jain *et al*. study^6^ and others^43,173^ spurred the development of several computational predictors,^5,40^ similar investigations using next-generation biotherapeutics will enable such methods to be refined, or new approaches to be developed, to yield accurate predictions of the developability profiles of these constructs.

### 4.3 Applications of artificial intelligence and machine learning toward antibody discovery, development, and manufacturing

Machine learning algorithms have been used for classification, regression, or clustering of biopharmaceutical experimental datasets. Machine learning models have been used for the prediction of protein secondary structure,^174,175^ relative solvent accessibility,^176–179^ protein folding,^180–183^ protein–protein interactions,^184–188^ and PTMs.^189–192^ Machine learning methods have also been applied to the prediction of aggregation using a classification tree ensemble with sequence-derived physicochemical properties.^7,193^ Other machine learning approaches such as gradient-boosting machines have been used for the prediction of CDR structure from protein sequence, particularly CDR-H3.^194,195^ The most common strategy used by these algorithms is the use of biophysical propensity scales as input features for machine learning methods to characterize the structural and functional properties of proteins.^196^

Narayanan *et al*. have reviewed the application of machine learning approaches in predicting the developability of antibody-based biologics.^197^ A machine learning algorithm has been shown to predict antibody developability solely by sequence using a dataset of 2400 antibodies.^58^ Here, a support vector machine model trained on physicochemical features with multiple sequence alignment emerged as the best machine learning pipeline combination to capture antibody developability from the sequence.

Deep learning approaches for antibody design and engineering are also becoming popular.^198^ Several deep learning models have been described for predicting paratope regions in antibody sequences,^199^ epitope-specific paratope identification,^200^ predicting antibody/antigen binding,^201^ CDR-H3 region optimization,^202^ and virtual screening for therapeutic antibody optimization.^203^ Deep learning algorithms offer the ability to capture key biophysical features and properties for any developability objective without the need to create complex theoretical functions. Consequently, deep learning approaches are ideal for cases where mechanistic understanding of the underlying developability issue is not fully understood. However, deep learning algorithms generally require large amounts of data, and so can be unsuitable for smaller datasets.

The choice of the machine learning algorithm is decided by the dataset availability and the objectives of the application. Supervised machine learning methods such as support vector machines, random forests, and conditional random fields are usually more appropriate for balanced datasets.^191^ Although machine learning-based methods lack the physical transparency of other approaches, their practical application is remarkably successful. Therefore, given that the amount of available training data across biological and structural databases is rapidly increasing, and that machine-learning algorithms are constantly improving, these methods are destined to play key roles in shaping the future of biopharmaceutical informatics.

## 5 Conclusion

The past two decades have seen transformational advances in the biomedical sciences. In particular, the Human Genome Project has triggered the development of NGS technologies, which are enriching biological databases with millions of sequences of proteins, including antibodies from myriad different sources. Furthermore, improvements in the pace and accuracy of protein structure determination techniques are contributing unprecedented amounts of high-quality structural data, comprising large numbers of antibody–antigen complexes.^204–206^ The increasing use of quantitative methods in biology has gradually transformed the way biological observations are made, and it is now possible to assemble large datasets of highly accurate measurements of antibody biophysical properties. Finally, computers able to perform complex calculations quickly are available, and extremely powerful algorithms for data mining and machine learning are constantly being developed. Taken together, these advances are enabling the antibody community to address questions that were essentially intractable a decade ago, including the development of highly accurate computational methods to streamline the development of biotherapeutics.

Here, we have described numerous metrics for computational developability assessment and established that no single tool or biophysical parameter can be used for predicting the developability potential of a biotherapeutic. The orthogonal combination of conceptually different algorithms should be used in developability assessment protocols to reduce method-specific biases. However, as stated by Narayaram *et al*.,^197^ “one common disadvantage of such *in silico* tools is that they use only protein sequences or structure-based information as input and usually do not consider the impact of formulation conditions”. The biophysical solution behavior is also influenced by the excipients and solution conditions of the formulated product. Therefore, the developability assessment algorithms will have more real-life practical applications if they also consider the solution conditions and formulation parameters in the algorithms. In addition, minimal information has been provided in the available literature on the validation of these tools in the industrial setting. Therefore, it is important that biopharmaceutical informatics approaches are uniformly applied across the industry to expand and accelerate their potential for biotherapeutics development.

Biopharmaceutical informatics can also be a valuable guide for the commercialization and licensing of antibody-based drugs. The insights from computational developability assessments can aid the due-diligence activities performed during licensing and acquisition transactions.^207^ The application of biopharmaceutical informatics tools is likely to increase in the future as new accurate and faster software are becoming available for generating antibody structure from the sequence for mAbs. Recently, AlphaFold,^208^ a neural-network-based algorithm that was recognized as the optimal solution to the protein-folding problem at the Critical Assessment of protein Structure Prediction (CASP) competition, has received wide media attention, but its efficacy in modeling antibodies remains unproven. The recent success of AlphaFold at predicting protein structures demonstrates the power of bioinformatics applications. With increasing efforts devoted to data curation and method development as described here, biopharmaceutical informatics holds the potential to play a leading role in selection and engineering of safe therapeutics.

## 6 Abbreviations

**Table:** 

ADC	Antibody–drug conjugate
ADR	Adverse drug reaction
APR	Aggregation-prone region
BCE	B-cell epitope
BCR	B-cell receptor
CDA	Computational developability assessment
CDR	Complementarity-determining region
HIC	Hydrophobic interaction chromatography
MHC	Major histocompatibility complex
NGS	Next-generation sequencing
PD	Pharmacodynamic
PDB	Protein data bank
PFA	Position frequency analysis
PK	Pharmacokinetic
PPV	Positive predictive value
PTM	Posttranslational modification
SAP	Spatial aggregation propensity
ScFv	Single-chain variable fragment
SEC	Size exclusion chromatography
TAP	Therapeutic antibody profiler
TCR	T-cell receptor
